# Expert Testimony

**Published:** 1903-03

**Authors:** 


### Expert Testimony.

THE meeting of the Medical Society of the County of Erie in January was of unusual importance. At that time the bench and the bar and the medical profession met to discuss the question which concerned all—medical expert testimony.

Judge Davie presented in a very able paper the faults and the merits of the present system of the employment of experts. He quoted from the opinions of different judges to show into what disrepute experts have fallen.

None of the quotations were more serious in their charges of bias than the opinion of Judge Davis, of the Supreme Court of Maine, in the Neil case:

If there is any kind of testimony that is not only of no value, but even worse than that, it is, in my judgment, that of medical experts. They may be able to state the diagnosis of a case more learnedly, but upon the questions whether it had at a given time reached a stage that the subject of it was incapable of making a contract or was irresponsible for his acts, the opinions of his neighbors, of men of good common sense, would be worth more than that of all the experts in the country.

The opinion of Lord Kenyon, is also important when he said:

Skilled witnesses come with such a bias on their minds to support the cause in which they are embarked that hardly any weight should be given their evidence.

The question of expert testimony is one which is most difficult to solve. Questions are constantly arising in the courts which can only be settled by expert opinions, and yet expert opinions cannot always be relied upon.

Expert opinions continually differ. In our judgment, experts in any field of thought will differ. They will differ in law as the decisions of the Court of Appeals will show. They will differ in matters of religion, politics and finance. They will necessarily differ on medical questions.

Nevertheless, we believe that experts would differ less frequently if they were given all the facts in a case, instead of a few well and skilfully chosen facts incorporated in a hypothetical question. Another cause, which can be eliminated, is entirely in the hands of the attorneys. If they really wish an honest opinion as to the merits of the case let them select men of acknowledged ability and reputation. Let them pursue this course instead of that other course, which is far too common, of seeking men who will testify to anything that may be desired.

We know of cases in which an attorney has brought the plaintiff to a physician's office and requested an examination. Upon that physician's report that the examination revealed only slight and temporary injuries, the attorney chose other, perhaps less skilful and less honest men, who swore stoutly to the incurable nature of the malady.

Such meetings as the one held by the Medical Society of the County of Erie are of value. It is important that lawyers and physicians should meet and discuss public questions which affect both professions and which affect the public. Let the leaders of both professions strive to devise a way which will make expert testimony important and not a commodity which can be bought and sold. We hope that the day may come when it will be permissible for a physician to testify that he examined the evidence and came to such an opinion, based upon the sworn testimony and upon the personal examination of the plaintiff or prisoner, as the case may be; that after he has given his opinion the expert should be asked how such an opinion is formed and that he be subjected to a rigid cross-examination. Such a method would avoid the use of the hypothetical question which never presents the truth, the whole truth, and nothing but the truth.

